# Evaluation of animal-to-human and human-to-human transmission of influenza A (H7N9) virus in China, 2013–15

**DOI:** 10.1038/s41598-017-17335-9

**Published:** 2018-01-11

**Authors:** Victor Virlogeux, Luzhao Feng, Tim K. Tsang, Hui Jiang, Vicky J. Fang, Ying Qin, Peng Wu, Xiling Wang, Jiandong Zheng, Eric H. Y. Lau, Zhibin Peng, Juan Yang, Benjamin J. Cowling, Hongjie Yu

**Affiliations:** 1 Department of Biology, Ecole Normale Supérieure de Lyon, Lyon, France; 2 Cancer Research Center of Lyon, UMR Inserm U1052, CNRS 5286 Lyon, France; 3 School of Public Health, Li Ka Shing Faculty of Medicine, The University of Hong Kong, Hong Kong Special Administrative Region, China; 4 Division of Infectious Disease, Key Laboratory of Surveillance and Early-Warning on Infectious Disease, Chinese Center for Disease Control and Prevention, Beijing, China; 5 School of Public Health, Fudan University, Key Laboratory of Public Health Safety, Ministry of Education, Xuhui District, Shanghai, 200032 China

## Abstract

A novel avian-origin influenza A(H7N9) virus emerged in China in March 2013 and by 27 September 2017 a total of 1533 laboratory-confirmed cases have been reported. Occurrences of animal-to-human and human-to-human transmission have been previously identified, and the force of human-to-human transmission is an important component of risk assessment. In this study, we constructed an ecological model to evaluate the animal-to-human and human-to-human transmission of H7N9 during the first three epidemic waves in spring 2013, winter/spring 2013–2014 and winter/spring 2014–2015 in China based on 149 laboratory-confirmed urban cases. Our analysis of patterns in incidence in major cities allowed us to estimate a mean incubation period in humans of 2.6 days (95% credibility interval, CrI: 1.4–3.1) and an effective reproduction number Re of 0.23 (95% CrI: 0.05–0.47) for the first wave, 0.16 (95% CrI: 0.01–0.41) for the second wave, and 0.16 (95% CrI: 0.01–0.45) for the third wave without a significant difference between waves. There was a significant decrease in the incidence of H7N9 cases after live poultry market closures in various major cities. Our analytic framework can be used for continued assessment of the risk of human to human transmission of A(H7N9) virus as human infections continue to occur in China.

## Introduction

Human infections with a novel avian influenza A (H7N9) virus were first reported in March 2013 in China, and by 27 September 2017 a total of 1533 laboratory-confirmed cases and 607 deaths have been officially reported^1^. A majority of H7N9 cases appear to have resulted from animal-to-human contact, particularly in live poultry markets (LPMs) in urban areas^2–5^. Temporary closure of LPMs was reported to effectively halt epidemics during spring 2013 in Shanghai, Nanjing, Hangzhou and Huzhou^3^ and during the second epidemic wave in the winter and spring of 2013–2014 in Shenzhen, Guangzhou, Hangzhou, Foshan and Ningbo using contact tracing data^2^. Although animal-to-human transmission is the major route of transmission, some previous studies identified a human-to-human transmission component using clusters of human infections^6^ and contact tracing data^7,8^ and found very low basic reproduction number estimates. On the other hand, Kucharski *et al*. estimated both the human-to-human transmission component and the animal-to-human transmission using ecological data and reported higher basic reproduction number estimates^9^. In this study, we aimed to evaluate transmission from animal-to-human and human-to-human using an ecological approach.

## Material and Methods

### Sources of data

Laboratory-confirmed cases of influenza A(H7N9) virus infection in China were reported to the Chinese Centres for Disease Control and Prevention, and information on these cases was recorded in a comprehensive database including case demographics, medical history, history of potential exposures, and clinical outcomes. The case definitions and laboratory test assays have been described previously^10,11^. In this study, we focused on cities with larger numbers of laboratory-confirmed cases (≥5 urban cases), and distinct dates of live poultry market closures, that would permit joint analysis of human infection dynamics and the impact of live poultry market closures. We therefore selected three cities during the first epidemic wave in spring 2013, namely Shanghai, Nanjing and Hangzhou^3^, five cities during the second epidemic wave in winter and spring of 2013–14, namely Shenzhen, Guangzhou, Hangzhou, Foshan and Ningbo^2^, and four cities during the third waves in winter and spring 2014–15, namely Shenzhen, Suzhou, Xiamen and Shanghai. In response to the rapid increase of cases in the first two waves, local authorities decided to close all LPMs during the first wave on April 6, 8 and 15, 2013 in Shanghai, Nanjing and Hangzhou respectively (Supplementary Table 1). Similarly, during the second wave in 2013–2014, all LPMs were closed in Ningbo, Shenzhen and Guangzhou on January 26, January 31 and February 15, respectively. In Foshan and Hangzhou, LPMs were progressively closed from February 7 to 13 and from January 21 to 24, respectively (Supplementary Table 1). During the third wave in 2014–2015, all LPMs were closed in Shenzhen and Shanghai on February 19 and in Suzhou and Xiamen, LPMs from a majority of districts were closed from January 12 (Supplementary Table 1).

### Statistical analysis

**Figure 1:**
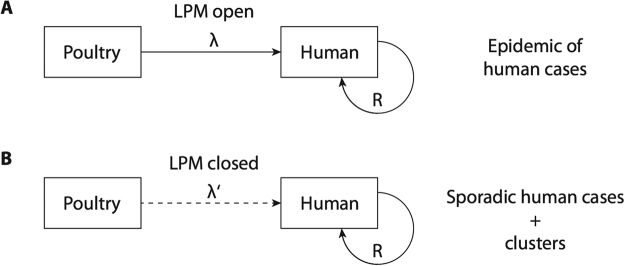
Conceptualisation of the modeling approach. Panel A shows the situation when LPMs are open, and H7N9 transmission from poultry to humans occurs at a rate λ, while human-to-human transmission also occurs with reproductive number Re. In this situation there may be an epidemic of human infections with H7N9. Panel B shows the situation when LPMs are closed, transmission from poultry to humans occurs at a reduced rate λ′, while human-to-human transmission also occurs with the same reproductive number Re as in panel A. In this situation there may be sporadic clusters of human infections with H7N9.

We developed a model that could evaluate both animal-to-human and human-to-human transmission, illustrated in Fig. 1. We first defined the animal-to-human transmission component based on exposure to live-poultry during LPM visits^12,13^, where the incidence of human infection was constant in each city before and after LPM closures with values λ and λ′ respectively. The effect of LPM closure could then be expressed in the form $(1-\frac{\lambda ^{\prime} }{\lambda })\times 100 \%$ indicating the proportionate reduction in incidence after LPM closures^3^. Separate data indicated a 95% reduction of the average number of individuals visiting LPM each day during both waves, respectively^14^. Because of the delay from infection to onset of symptoms, following the incubation period distribution, some human cases might only have symptom onset after the LPM closures despite infection occurring before the closure. We therefore included the incubation period distribution in our model, assuming that it followed a Weibull distribution as previously reported (see Appendix)^15^. We included the possibility of human-to-human transmission following a likelihood-based method for estimating the effective reproduction number Re, i.e the average number of secondary cases per infectious case in a population made up of both susceptible and non-susceptible hosts^16^. We assumed that the serial interval, i.e the time between onset of symptoms in an index case and a secondary case, of H7N9 followed a Poisson distribution with a mean of 7.5 days in the main analysis, and we tested a range of values between 5.5 and 9.5 days in the sensitivity analysis (see Appendix)^7^. We simultaneously estimated the animal-to-human force of infection, i.e rate at which susceptible individuals acquire an infection, in each city during the different waves before and after LPM closure as well as the incubation period distribution, that we supposed to be constant over time and location, and the human-to-human transmission component with the effective reproduction number Re using a Poisson likelihood approach based on the time series data available for each wave (see Appendix). Parameter estimation was conducted using a Monte Carlo Markov Chain (MCMC) method in a Bayesian framework. For each parameter, we drew 10,000 samples from the posterior distribution after a burn-in of 5,000 iterations. Convergence of MCMC chains were evaluated using Geweke’s diagnostic test^17^. All reported estimates are based on the posterior probability distribution of each parameter estimated with the MCMC process and we therefore provide corresponding credibility intervals based on the posterior distribution for each estimate considering the Bayesian framework. All analyses presented here were conducted using R version 3.2.2 (R foundation for Statistical Computing, Vienna, Austria).

## Results

**Figure 2:**
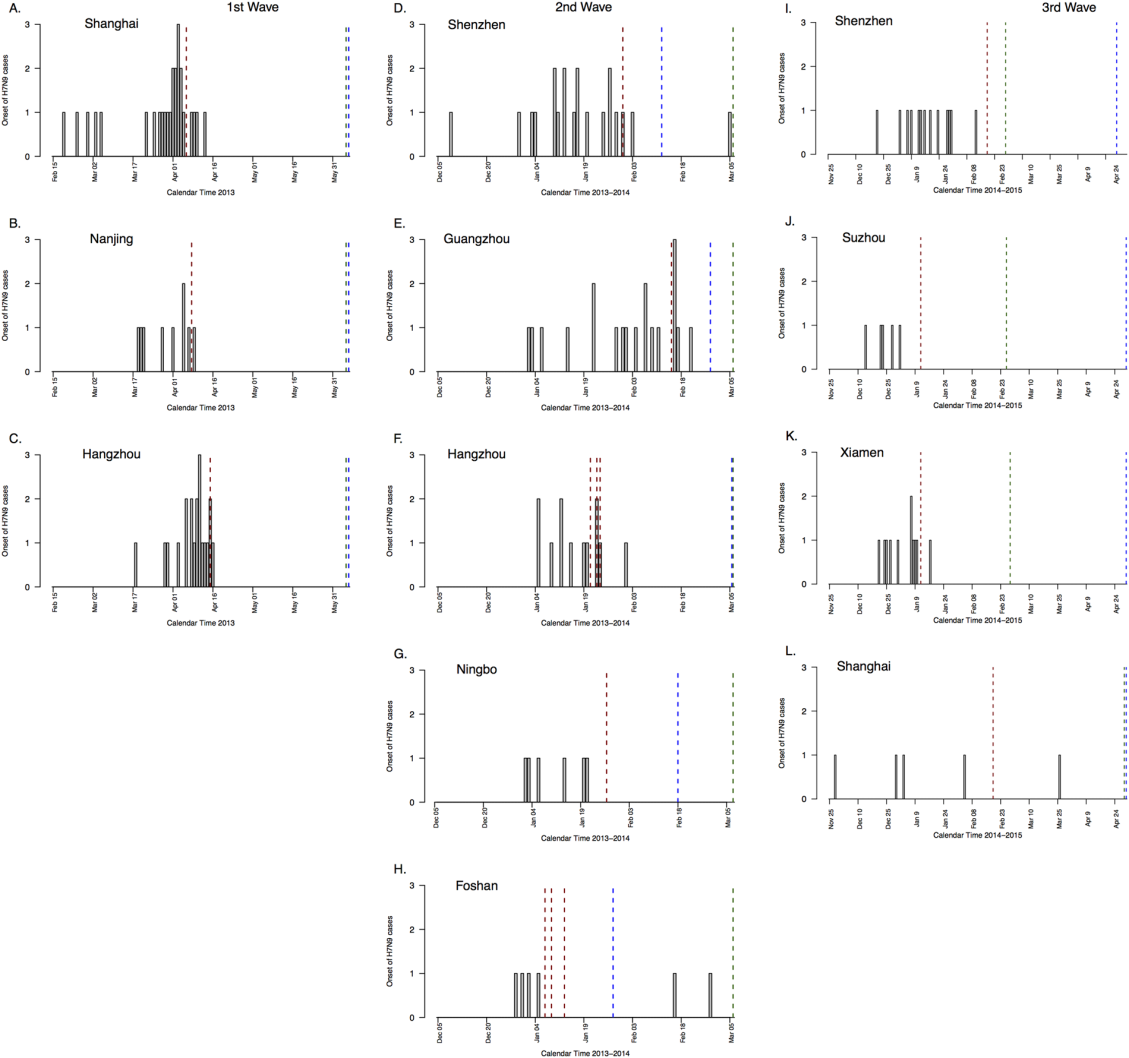
Dates of influenza A(H7N9) urban cases and LPM closures in Shanghai, Nanjing and Hangzhou during the first wave (February 2013 – June 2013), in Shenzhen, Guangzhou, Hangzhou, Ningbo and Foshan area during the second wave (December 2013 – March 2014) and in Shenzhen, Suzhou, Xiamen and Shanghai area during the third wave (November 2014 – May 2015). The grey bar for each day indicates the number of laboratory-confirmed cases with onsets on that day. Red vertical lines indicate the dates of closures of live poultry markets in each area (markets in Guangzhou and Foshan areas were closed on different dates during the second wave), blue vertical lines indicate the last date used for each area in analyses and green vertical lines indicate the last day of time horizon.

We included a total of 55 laboratory-confirmed urban cases in the first wave, 60 laboratory-confirmed urban cases in the second wave and 34 laboratory-confirmed urban cases in the third wave, in 3, 5 and 4 cities respectively. The dates of illness onset of these 149 cases in 9 cities are shown in Fig. 2. The closure dates are indicated by dotted vertical lines in Fig. 2, and in each city there is a clear drop in the incidence rate of new cases shortly after the implementation of market closures.

**Figure 3:**
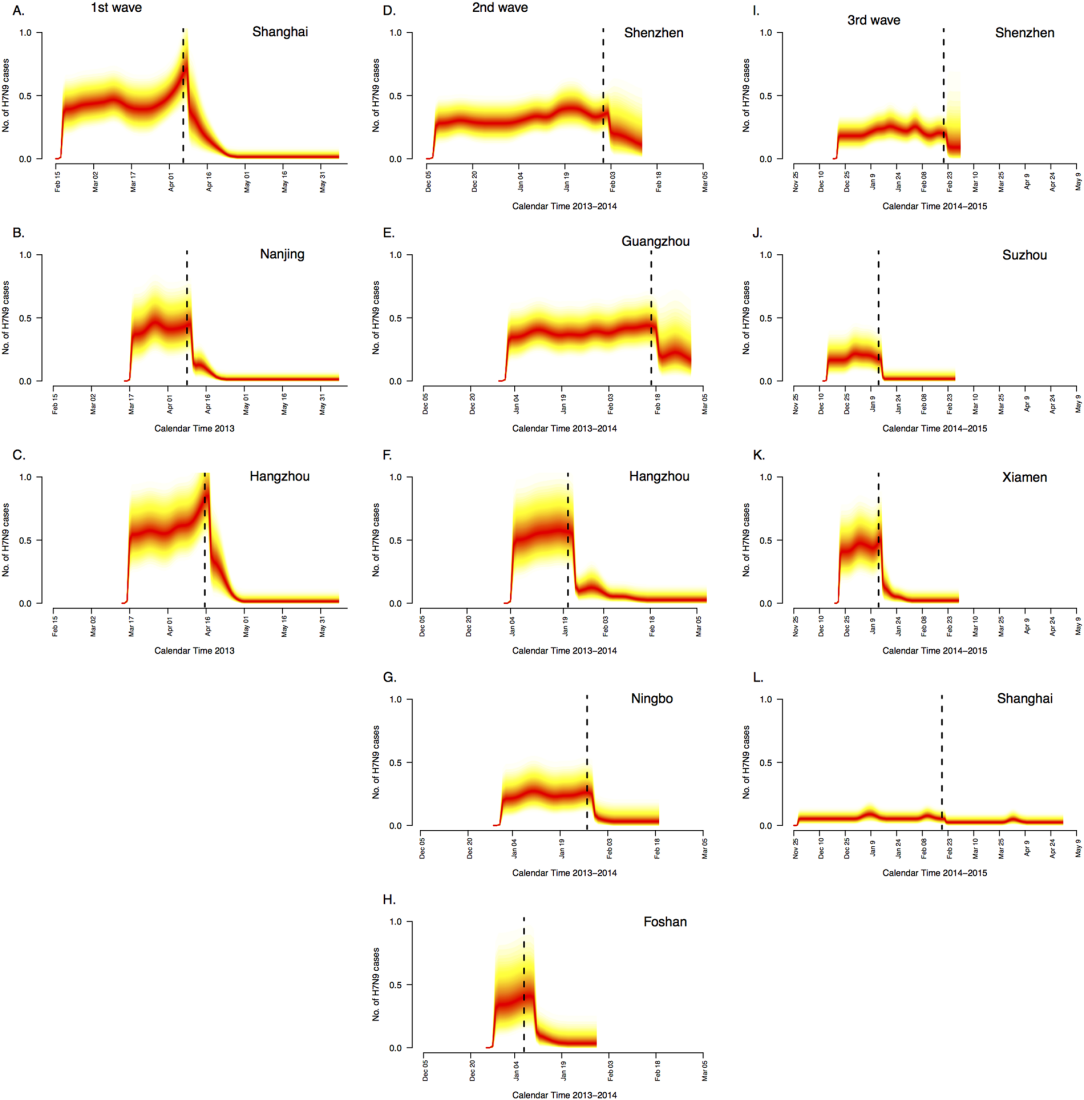
Posterior estimates of the mean daily number of illness onsets of A(H7N9) cases during the first wave in Shanghai, Nanjing and Hangzhou area, the second wave in Shenzhen, Guangzhou, Hangzhou, Ningbo and Foshan area and the third wave in Shenzhen, Suzhou, Xiamen and Shanghai. Darker colors indicate regions with higher posterior density on a given day. Black vertical lines indicate the dates of closures of live poultry markets in each area (markets in Guangzhou and Foshan areas were closed on different dates during the second wave).

We estimated the force of animal-to-human transmission and the force of human-to-human transmission in each city. We found that closures of LPMs in the different waves were associated with significant decreases in incidence rates in each city (Fig. 3). Regarding animal-to-human transmission, the median of the posterior estimates of the reduction in incidence rate after closure of LPMS were 95%, 95% and 96% in Shanghai, Nanjing and Hangzhou with 95% credibility interval included between 89% and 100% (Table 1). During the second and the third waves, the median of the posterior estimates of incidence rate reduction had generally lower point estimates with wider credibility intervals (Table 1). We also simultaneously estimated the incubation period distribution of H7N9 across all locations and found a mean incubation period of 2.6 days (95% CrI: 1.4–3.1).

**Table 1:** Parameter estimates of incidence rates before and after live poultry market closures among urban cases.

Parameters	Expected daily number of infections before closure	Expected daily number of infections after closure	Reduction in mean daily number of infections^1^	Re (95% CrI)
**First epidemic wave (Spring 2013)**				
Shanghai	0.40 (0.21–0.62)	0.02 (0.00–0.07)	95% (89–100)	0.23 (0.05–0.47)
Nanjing	0.38 (0.17–0.67)	0.02 (0.00–0.06)	95% (90–100)	
Hangzhou	0.55 (0.28–0.87)	0.02 (0.00–0.08)	96% (90–100)	
**Second epidemic wave (2013–2014)**				
Shenzhen	0.28 (0.13–0.46)	0.13 (0.00–0.43)	56% (6–98)	0.16 (0.01–0.41)
Guangzhou	0.35 (0.18–0.56)	0.15 (0.01–0.48)	58% (14–97)	
Hangzhou^2^	0.50 (0.23–0.85)	0.03 (0.00–0.12)	93% (86–100)	
Ningbo	0.22 (0.08–0.43)	0.05 (0.00–0.17)	79% (61–98)	
Foshan^3^	0.31 (0.08–0.71)	0.06 (0.00–0.23)	80% (68–98)	
**Third epidemic wave (2014–2015)**				
Shenzhen	0.19 (0.08–0.32)	0.13 (0.00–0.49)	28% (−53–95)	0.16 (0.01–0.45)
Suzhou	0.18 (0.06–0.35)	0.02 (0.00–0.09)	86% (74–99)	
Xiamen	0.42 (0.20–0.71)	0.03 (0.00–0.10)	93% (86–100)	
Shanghai	0.06 (0.02–0.12)	0.03 (0.00–0.08)	48% (32–79)	
Mean Incubation Period (95% CrI)		2.6 (1.4–3.1)		

^1^The ratio (1 − λ′/λ) × 100% in a specific city reflected the local impact of LPM closure in reducing mean daily number of infections.

^2^Three different LPM closure dates were considered for this area, ie. 21 Jan 2014, 23 Jan 2014 and 24 Jan 2014.

^3^Two different LPM closure dates were considered for this area, i.e 7 Feb 2014 and 13 Feb 2014.

The relative contribution of human-to-human transmission was evaluated by estimating the reproduction number in each epidemic wave, assuming in the main analysis a mean serial interval of 7.5 days. During the first wave, we estimated Re to be 0.23 (95% CrI: 0.05–0.47) whereas during the second and third waves we estimated Re to be 0.16 (95% CrI: 0.01–0.41) and 0.16 (95% CrI: 0.01–0.45) respectively. There was no statistically significant difference between the different estimates of Re.

As a sensitivity analysis, we estimated Re using alternative values for the mean serial interval between 5.5 and 9.5 days (Supplementary Table 2). No significant differences between the two waves were observed, although Re tends to increase when the mean serial interval decreases, particularly for the first wave.

We also fitted the model including 11, 29 and 3 semi-urban cases that were reported during the first, second and third wave in the different cities, respectively (Supplementary Table 3). No clear difference with the main analysis was observed regarding the reduction of animal-to-human transmission and a slightly but not-significant higher basic reproduction number was observed in the first two waves but not in the third wave (first wave: Re = 0.26; 95% CrI: 0.08–0.49; second wave: Re = 0.24; 95% CrI: 0.05–0.48 and third wave: Re = 0.13; 95% CrI: 0.00–0.39).

To assess the effect of potential unreported primary cases on the reproduction number, we also fitted the model to simulated epidemics derived from the ecological data we collected and we considered several rates (20%, 40% and 60%) of unreported cases (see Appendix). We observed a decrease of the reproduction number in the several waves when the rate of unreported primary cases increased (Supplementary Table 4).

## Discussion

Our findings show that LPM closures were associated with significant decreases in the incidence of animal-to-human transmission in the different cities considered in this study, consistent with previous reports^2,3^. We found evidence of a low risk of human-to-human transmission, with an estimated reproductive number far below 1 (Table 1). Our estimates of the reproduction number are slightly higher to those reported in a cluster analysis, i.e. considering only the human-to-human transmission component, during the first two waves of H7N9 cases where the authors estimated a mean basic reproduction number R_e_ to be 0.08 (95% CI: 0.05–0.13) and estimates range from 0.07 to 0.12^7^. On the other hand, our estimates are slightly lower than those estimated in a study conducted by Kucharski *et al*.^9^, who used a model that incorporated animal-to-human and human-to-human transmission and reported a significant increase of the basic reproduction number R_0_ in Zhejiang province in the second wave (R_0_ = 0.35; 95% CrI: 0.15–0.65) compared with the first wave (R_0_ = 0.06; 95% CrI: 0.00–0.25). We did not observe such a pattern in our study between the first and the second wave of H7N9 cases, although we considered a constant reproduction number Re over the different locations during each wave. Indeed, strict selection of cases and more particularly of urban cases is crucial to fit the condition of our model that is based on the visits of LPMs located in the cities, which explains why the number of considered cases in the main analysis of our study is lower than the one reported in their study and which could explain why the estimates are different (see Appendix)^9^. Incorporating cases from the outskirts of cities, where human exposure risk was generally lower but did not change dramatically after LPM closures, would have led to misattribution of some cases of animal-to-human transmission as human-to-human transmission after LPM closures.

A strength of our model is that we were able to estimate simultaneously the relative contributions of animal-to-human and human-to-human transmission components using the impact of LPM closure. To obtain the best fit with the observed time series, we integrated a crucial epidemiological parameter which is the incubation period into the animal-to-human transmission model (as the incubation period is already considered in the serial interval of the human-to-human transmission model)^3,15^. We consequently estimated the incubation period in the same time than the animal-to-human transmission in order to take into account the cases that were reported after the LPMs closure but were infected before due to the incubation period. Not considering the delayed onset of these few cases after LPM closure could have led to an overestimation of human-to-human transmission. Our estimates are similar to those reported in previous studies based on exposure data and estimating the incubation period distribution with parametric methods^2,3,8,15^. Our ecological model slightly differs with cluster-based analysis in the term that this latter type of analysis is based on the assumption that each cluster is the result of human-to-human transmission starting from a single index case. Most of cluster models allow for uncertainty in case detection in the close contact investigation, however they often do not consider the possibility of coexposure of epidemiologically linked cases to the same source of zoonotic/environmental infection, which could lead to overestimation.

We considered a mean serial interval of 7.5 days in our main analysis based on a previous analysis of household transmission^7^. In our sensitivity analyses we showed that the basic reproduction number is inversely proportional to the mean serial interval, particularly during the second wave, which highlights the need of accurate estimates for this parameter.

When we accounted for the potential under-ascertainment of cases, the reproductive number estimates were somewhat lower (Supplementary Table 4). A continuing priority in avian influenza epidemiology is assessment of the proportion of infections that are ascertained^18,19^. Mild infections are occasionally reported, and it is likely that many mild infections are never identified and laboratory-confirmed, skewing our impression of the severity of typical influenza A(H7N9) infections in humans^20^. Under-ascertainment of infections has been a limitation of epidemiologic studies of H7N9 to date, and large serologic studies would be valuable because they could indicate the cumulative incidence of human infections and therefore the degree of under-ascertainment^21^.

Our estimates of the reproduction number for the different waves show that the human-to-human transmission component of H7N9 appears to be relatively low and not sustainable. An Influenza Risk Assessment Tool (IRAT) has recently been developed by the CDC in order to estimate the potential pandemic risk posed by influenza A viruses based on ten different criteria related to the properties, the attributes of the population and the ecology and epidemiology characteristics of the virus^22^. Using this tool, the CDC characterized influenza H7N9 as the virus with the highest potential pandemic risk among all influenza viruses, more particularly if the virus achieves in the future sustainable human-to-human transmission. However until now, the different waves caused by H7N9 have been characterized by low human-to-human transmission^23^.

This study has some limitations. First, we assumed that the reproduction number Re was constant over location during each wave due to the low number of urban cases reported in the different locations, in order to obtain accurate posterior estimates of this parameter using larger time series data. Second, we could not estimate the serial interval distribution which influences the value of the basic reproduction number in our model. Third, we made the simple assumption of a constant force of infection before and after LPM closure in the market hazard model in each city. Despite these limitations, we were able to estimate the human-to-human transmission component using an ecological model that took into account the incubation period distribution, which explains partially the observed pattern of new H7N9 cases after LPM closure.

In conclusion, LPM closure was an efficient intervention measure to decrease the daily number of H7N9 cases during both waves, as human-to-human transmission occurred only sporadically. Nevertheless, LPM closures are a drastic measure, and other more sustainable interventions may be valuable in the longer term to protect animal and human health against avian influenza viruses. The analytic framework we have described here should be useful for continued assessment of the risk of H7N9 by permitting monitoring of the human-to-human transmissibility.

## Electronic supplementary material

Appendix
