# Supplementary material for: Evaluation of animal-to-human and human-to-human transmission of influenza A (H7N9) virus in China, 2013–15
Source: Sci Rep. 2018 Jan 11;8:552. doi: 10.1038/s41598-017-17335-9 (PMC5765021; doi:10.1038/s41598-017-17335-9)
Supplement: Supplementary file 1 — Appendix [file 41598_2017_17335_MOESM1_ESM.pdf]

Appendix

Evaluation of animal-to-human and human-to-human transmission of influenza A (H7N9) virus in China, 2013–15

Victor Virlogeux[1,2,3], Luzhao Feng[4], Tim K. Tsang[3], Hui Jiang[4], Vicky J. Fang[3], Ying Qin[4], Peng Wu[3], Xiling Wang[5], Jiandong Zheng[4], Eric H. Y. Lau[3], Zhibin Peng[4], Juan Yang[4], Benjamin J. Cowling[3], Hongjie Yu[5]

Victor Virlogeux and Luzhao Feng are joint first authors.

Benjamin J. Cowling and Hongjie Yu are joint senior authors.

Affiliations:

1. Department of Biology, Ecole Normale Supérieure de Lyon, Lyon, France.

2. Cancer Research Center of Lyon, UMR Inserm U1052, CNRS 5286, Lyon, France.

3. School of Public Health, Li Ka Shing Faculty of Medicine, The University of Hong Kong, Hong Kong Special Administrative Region, China.

4. Division of Infectious Disease, Key Laboratory of Surveillance and Early-Warning on Infectious Disease, Chinese Center for Disease Control and Prevention, Beijing, China.

5. School of Public Health, Fudan University, Key Laboratory of Public Health Safety, Ministry of Education, Xuhui District, Shanghai 200032, China

Corresponding author:

Hongjie Yu, MD PhD, School of Public Health, Fudan University, Key Laboratory of Public Health Safety, Ministry of Education, Xuhui District, Shanghai 200032, China.

cfetpyhj@vip.sina.com, +86 21 54237628 (Tel); +86 21 54237628 (fax)

Market hazard model: animal-to-human transmission

First we defined:

- $N_{pre,t,i}$ is the average number of people who visited LPMs in area *i* before closure.

- $N_{ost,t,i}$ is the average number of people who visited LPM in area *i* after closure (95% reduction of visits number was considered).

- $\lambda_{pre,i}$ is the constant force of infection in the area *i* in LPM before closure

- $\lambda_{post,i}$ is the constant force of infection in the area *i* in LPM after closure.

- F(t) is the CDF of the incubation period of H7N9 following a Weibull distribution with parameters (a,b).

- A_i is the date on which the first case was anounced in area *i*.

- B_i = Ai – 4 and T_i are the start and end of time horizon for area *i*.

The 4 days adjustment was made to account for any potential errors associated with variations in symptoms definition, patient recall of symptoms onset and incubation period.

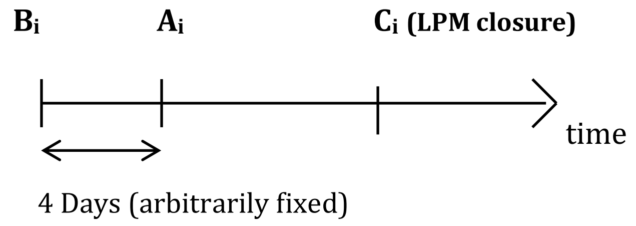

- $X_{t,i}$ is the number of confirmed cases with onset on day *t* in area *i.*

- $C_{i,j}$ is the date of LPM closure in area *i*.

We assumed that the population visiting LPM in each area *i* was subject to a daily per capita force of infection $\lambda_{pre,i}$ before any live poultry market (LPM) was closed and $\lambda_{post,i}$ after all LPMs were closed.

New infections in area *i* occurred according to a Poisson process such that the number of infections on day *t* was Poisson distributed with mean $\lambda_{pre,i} N_{pre,t,i}$ for $t \in [Ai, Ci - 1]$ and $\lambda_{post,i} N_{post,t,i}$ for $t \in [Ci, T]$.

We assumed that the incubation period followed the same (cumulative) probability Weibull distribution F with scale *a* and shape *b* for all cities.

Under these assumptions, the number of cases with onset on day t in area *i* was Poisson distributed with mean :

$$h_{a,i}(t) = \begin{cases} F(t) \times N_{pre,i} \times \lambda_{pre,i} & \text{for } A_i \leq t \leq C_i - 1 \\ F(t - C_i) \times N_{post,i} \times \lambda_{post,i} + (1 - F(t - C_i)) \times N_{pre,i} \times \lambda_{pre,i} & \text{for } C_i \leq t \leq T_i \end{cases}$$

Human-to-human transmission model:

We defined the human-to-human transmission, assuming that an infected individual has an infectiousness profile, i.e. the serial interval of H7N9, following a Poisson distribution with mean S_p. Using the effective reproduction number Re, i.e. the number of new infections generated by each infectious individual, we defined $h_H(t)$ the expected number of new human cases with onset on day *t* in area *i*:

$$h_{H,i}(t) = \sum_{j=1}^{\min(k,t)} R_e N(t - j + 1) \frac{{S_p}^j e^{-S_p}}{j!} \quad for\ t \geq 1$$

where :

- k is the maximum value of the serial interval (we arbitrarily fixed k=14 days).

- N(t) is the number of newly infected individuals at time t.

- S_p is the mean serial interval.

- Re is the effective reproduction number assumed to be constant over time and in the different areas.

The likelihood for the time series of observed human cases in all the LPM observed in this study was defined using the PDF of Poisson distribution :

$$L\begin{pmatrix} \lambda_{pre,SHA}, \lambda_{post,SHA}, \lambda_{pre,NA}, \lambda_{post,NA}, \lambda_{pre,HAf}, \lambda_{post,HAf} \lambda_{pre,SH}, \lambda_{post,SH}, \lambda_{pre,GU}, \lambda_{post,GU}, \lambda_{pre,FO} \\ , \lambda_{post,FO}, \lambda_{pre,NI}, \lambda_{post,NI}, \lambda_{pre,HAs}, \lambda_{post,HAs}, \lambda_{pre,SHE3}, \lambda_{post,SHE3}, \\ \lambda_{pre,SU}, \lambda_{post,SU}, \lambda_{pre,XI}, \lambda_{post,XI}, \lambda_{pre,SHA3}, \lambda_{post,SHA3}, a, b, R_e \end{pmatrix} = \prod_{i \in (SHA,NA,HAf,SH,GU,FO,NI,HAs,SHE3,SU,XI,SHA3)} \prod_{t=A_i}^{T_i} \frac{h_i(t)^{X_{t,i}} e^{-h_i(t)}}{X_{t,i}!}$$

where $\mathbf{h_i(t)=h_{a,i}(t)+h_{H,i}(t)}$**,** the sum of animal-to-human and human-to-human H7N9 cases at each day t.

We estimated the pre- and post-LPM closure constant force of infection, the parameters of the incubation period distribution and Re by fitting the model to the epidemic curve data in the different areas using MCMC methods.

We assumed a semi-informative prior (normal distribution with mean 0 and standard deviation 10) for a and non-informative flat priors for all other parameters. We drew 10,000 samples from the posterior distributions after a burn-in of 5,000 iterations.

Sensitivity analysis

To assess the impact of unreported primary cases, we simulated epidemics based on the ecological data we collected and on the assumption that on a given day, the number of reported cases is defined as: $M_t = qN_t$, where q is the proportion of reported cases, M_t the number of reported cases and N_t the real number of cases each given day. Under this

assumption, we simulated epidemics by adding the ecological data with the number of unreported cases estimated each day, using a binomial distribution with parameters *p*, i.e the probability of unreporting (1-q) and *n* the number of reported cases that given day. We simulated 1,000 epidemics for each value of *p* and we fitted the model to each of this simulated epidemic. We finally merged all the posterior distributions to get the estimates of each parameter.

Example R syntax for estimating H7N9 animal-to-human and human-to-human transmission

```
# Upload the serial interval data for each location

#First wave of H7N9 cases
serial_data_Shanghai<-read.csv("")
serial_data_Nanjing<-read.csv("")
serial_data_Hangzhou_f<-read.csv("")

#Second wave of H7N9 cases
serial_data_Shenzhen<-read.csv("")
serial_data_Guangzhou<-read.csv("")
serial_data_Hangzhou_s<-read.csv("")
serial_data_Ningbo<-read.csv("")
serial_data_Foshan<-read.csv("")

#Indicate the LPM closure dates

#First wave of H7N9 cases
fermeture_Shanghai<-as.Date()
fermeture_Nanjing<-as.Date()
fermeture_Hangzhou_f<-as.Date()

#Second wave of H7N9 cases
fermeture_Shenzhen<-as.Date()
fermeture_Guangzhou<-as.Date()
fermeture_Hangzhou<-as.Date()
fermeture_Ningbo<-as.Date()
fermeture_Foshan<-as.Date()

#Indicate the number of individuals visiting LPM before closure
Reduction=0.05
 #First wave of H7N9 cases
N_pre_Shanghai=c()  ; N_post_Shanghai=Reduction*N_pre_Shanghai
N_pre_Nanjing= c() ; N_post_Nanjing=Reduction*N_pre_Nanjing
N_pre_Hangzhou_f=c()  ;  N_post_Hangzhou_f=Reduction*N_pre_Hangzhou_f
#Second wave of H7N9 cases
N_pre_Shenzhen=c()  ; N_post_Shenzhen=Reduction*N_pre_Shenzhen
N_pre_Guangzhou=c()  ; N_post_Guangzhou=Reduction*N_pre_Guangzhou
```

```
N_pre_Hangzhou_s=c()  ; N_post_Hangzhou_s=Reduction*N_pre_Hangzhou_s
N_pre_Ningbo=c()  ; N_post_Ningbo=Reduction*N_pre_Ningbo
N_pre_Foshan=c()  ; N_post_Foshan=Reduction*N_pre_Foshan

#Function to estimate the number of cases from animal-to-human transmission
animal_to_human<- function(t,lambda_pre,lambda_post,N_pre,N_post,Date_of_closure,a,b)
 {tot=tot1=tot2=c()
 t1=t[t<Date_of_closure]
 t2=t[t>Date_of_closure-1]
 tot1=lambda_pre*N_pre*pweibull(t1,a,b)
 tot2=lambda_pre*N_pre*(1-pweibull(t2-Date_of_closure,a,b))+lambda_post*N_post*pweibull(t2-
 Date_of_closure,a,b)
 return(tot=list(tot1,tot2))
 }

 #Function to estimate the number of cases from human_to_human transmission
 human_to_human<-function(t,R,mean_serial_interval,serial_data)
 {
   retour=c(0,0,0,0,0,rep(NA,length(t)-5))
   trans=c(NA)
   for (j in 6:length(t)) {
     trans=sum(R*serial_data[seq.int(t[j],t[j]-min(max_serial_interval,t[j]-
 4)+1)]*dpois(seq_len(min(max_serial_interval,t[j]-4)),mean_serial_interval))
     retour[j]=trans}
   return(list((retour)))
 }

  #Likelihood function
  likelihood=c()
  likelihoodfn<-function(lambda_pre,lambda_post,N_pre,N_post,Date_of_closure,serial_data,a,b,R)
  {
   t=c(0,seq_len(length(serial_data)-1))
   tbis=t+1
likelihood[tbis]=dpois(serial_data[tbis],unlist(animal_to_human(t,lambda_pre,lambda_post,N_pre,
 N_post,Date_of_closure,a,b))+unlist(human_to_human(t,R,mean_serial_interval,serial_data)))
   return(sum(log(likelihood)))
 }
```

Supplementary Table 1. Live poultry markets dates of closure, officially first reported case and time horizon in each location during three waves of H7N9 (2013-2014)

Area	Date of LPM closure	Date of the officially first reported case	End of time horizon
First wave			
Shanghai (SHA1)	06 Apr 2013	29 Mar 2013	07 Jun 2013
Nanjing (NA)	08 Apr 2013	30 Mar 2013	07 Jun 2013
Hangzhou (HAf)	15 Apr 2013	03 Apr 2013	07 Jun 2013
Second wave			
Shenzhen (SHE2)	31 Jan 2014	09 Dec 2013	13 Feb 2014
Guangzhou (GU)	15 Feb 2014	02 Jan 2014	28 Feb 2014
Nanhai district in Foshan (FO)[3]	7 Feb 2014<br>13 Feb 2014	25 Dec 2013	29 Jan 2014
Ningbo (NI)[1]	26 Jan 2014	01 Jan 2014	18 Feb 2014
Hangzhou (HAs)[2,3]	21 Jan 2014<br>23 Jan 2014<br>24 Jan 2014	02 Jan 2014	07 Mar 2014
Third wave			
Shenzhen (SHE3)	19 Feb 2015	20 Dec 2014	01 Mar 2015
Suzhou (SU)[4]	12 Jan 2015	14 Dec 2014	26 Feb 2015
Xiamen (XI)[5]	12 Jan 2015	21 Dec 2014	28 Feb 2015
Shanghai (SHA3)	19 Feb 2015	28 Nov 2014	01 May 2015

[1] *Four districts were considered in Ningbo downtown: Haishu, Jiangdong, Jiangbei and Yinzhou*
[2] *Hangzhou downtown area (Gongshu, Shangcheng, Xiacheng, Jianggan, Xihu and Binjiang) and two suburbans areas (Xiaoshan and Yuhang) were considered.*
[3] *several LPM closure dates were reported for these areas.*
[4] *For Suzhou area, only LPMs in Wuzhong, Xiangcheng, Gusu, Suzhou Industry Zone, and Suzhou High and New Technology Development Zone were closed*
[5] *For Xiamen area, only LPMs with reported H7N9 cases and LPMs from Simin district were closed*

Supplementary Table 2. Estimates of the reproduction number during the first, second and third wave of H7N9 cases using different mean serial interval values.

	Reproduction number Re (95% CrI)		
**Mean serial interval (S)**	**First wave**	**Second wave**	**Third wave**
S=5.5 days	0.38 (0.13 – 0.68)	0.16 (0.01 – 0.41)	0.17 (0.01 – 0.46)
S=6.5 days	0.30 (0.09 – 0.58)	0.17 (0.01 – 0.43)	0.16 (0.01 – 0.47)
S=7.5 days	0.23 (0.05 – 0.47)	0.16 (0.01 – 0.41)	0.16 (0.01 – 0.45)
S=8.5 days	0.19 (0.04 – 0.39)	0.16 (0.01 – 0.40)	0.16 (0.01 – 0.42)
S=9.5 days	0.16 (0.02 – 0.37)	0.15 (0.01 – 0.41)	0.16 (0.01 – 0.45)

Supplementary Table 3. Parameter estimates of incidence rates before and after live poultry market closures among all cases (i.e including rural cases) during the first, second and third waves.

Parameters	Expected daily number of infections before closure	Expected daily number of infections after closure	Reduction in mean daily number of infections[1]	Re (95% CrI)
**First epidemic wave (Spring 2013)**				
Shanghai	0.42 (0.23 – 0.66)	0.01 (0.00 – 0.07)	95% (89 – 100)	
Nanjing	0.37 (0.15 – 0.68)	0.02 (0.00 – 0.08)	94% (88 – 100)	0.26 (0.08 – 0.49)
Hangzhou	0.71 (0.40 – 1.07)	0.02 (0.00 – 0.07)	97% (93 – 100)	
**Second epidemic wave (2013-2014)**				
Shenzhen	0.27 (0.12 – 0.45)	0.11 (0.00 – 0.39)	60% (14 – 98)	
Guangzhou	0.34 (0.16 – 0.56)	0.19 (0.01 – 0.62)	43% (-11 – 96)	
Hangzhou[2]	0.90 (0.49 – 1.40)	0.05 (0.00 – 0.20)	94% (86 – 100)	0.24 (0.05 – 0.48)
Ningbo	0.32 (0.13 – 0.59)	0.05 (0.00 – 0.20)	84% (66 – 99)	
Foshan[3]	0.42 (0.14 – 0.85)	0.06 (0.00 – 0.21)	86% (76 – 99)	
**Third epidemic wave (2014-2015)**				
Shenzhen	0.19 (0.09 – 0.33)	0.14 (0.00 – 0.50)	31% (-53 – 96)	
Suzhou	0.18 (0.06 – 0.35)	0.02 (0.00 – 0.09)	86% (74 – 99)	0.13 (0.00 – 0.39)
Xiamen	0.46 (0.22 – 0.76)	0.06 (0.01 – 0.15)	88% (81 – 97)	
Shanghai	0.06 (0.02 – 0.12)	0.03 (0.00 – 0.08)	47% (32 – 83)	
Mean Incubation Period (95% CrI)		3.1 (2.0 – 4.0)		

[1] The ratio λ post/ λ pre in a specific city reflected the local impact of LPM closure in reducing mean daily number of infections.

[2]Three different LPM closure dates were considered for this area, ie. 21 Jan 2014, 23 Jan 2014 and 24 Jan 2014.

[3]Two different LPM closure dates were considered for this area, ie. 07 Feb 2014 and 13 Feb 2014.

Supplementary Table 4. Estimates of the reproduction number during the first, second and third wave of H7N9 using different proportions of unreported cases.

	Reproduction number Re[1] (95% CrI)		
**Proportion of unreported cases (p)**	**First wave**	**Second wave**	**Third wave**
0%	0.23 (0.05 – 0.47)	0.16 (0.01 – 0.41)	0.16 (0.01, 0.45)
20%	0.21 (0.05 – 0.43)	0.16 (0.01 – 0.39)	0.15 (0.01, 0.42)
40%	0.23 (0.07 – 0.46)	0.15 (0.01 – 0.35)	0.13 (0.01, 0.36)
60%	0.20 (0.06 – 0.40)	0.14 (0.01 – 0.34)	0.13 (0.01, 0.35)

[1]The reproduction number was estimated by fitting the model to 1,000 simulated epidemics for different proportion of unreported cases
